# Professor Klaus Fassbender: The Father of Mobile Stroke Units

**DOI:** 10.7759/cureus.69050

**Published:** 2024-09-10

**Authors:** Anna Podlasek, Silke Walter, Radim Licenik, Iris Grunwald

**Affiliations:** 1 Image Guided Therapy Research Facility (IGTRF), University of Dundee, Dundee, GBR; 2 Tayside Innovation Medtech Ecosystem (TIME), University of Dundee, Dundee, GBR; 3 Nottingham Biomedical Research Centre, University of Nottingham, Nottingham, GBR; 4 Department of Neurology, Saarland University Clinic, Homburg, DEU; 5 Acute Stroke Centre, North West Anglia NHS Foundation Trust, Peterborough, GBR; 6 Zlin Regional, Emergency Medical Services, Zlin, CZE

**Keywords:** alzheimer, fassbender, mobile stroke unit, pioneer, stroke

## Abstract

Professor Klaus Fassbender is a distinguished neurologist from Germany, widely recognized for his groundbreaking contributions to the fields of neurology and neurodegenerative disease. His work has been pivotal in advancing our understanding of the pathophysiological mechanisms underlying neurodegenerative disorders, including Alzheimer's and Parkinson's disease, as well as in refining therapeutic strategies for their treatment. His studies in cerebrovascular disease have elucidated the complex molecular and cellular processes involved in ischemic and hemorrhagic stroke, leading to the development of novel therapeutic interventions, often bridging the gap between laboratory discoveries and their application in clinical settings. Professor Klaus Fassbender is “the father” of the mobile stroke unit (MSU). With the “time is brain” concept in mind, he proposed and developed the MSU concept for the first time, allowing prehospital stroke imaging, diagnosis, and treatment directly at the site of emergency. This concept reduced times between symptoms onset and treatment, resulting in an increased proportion of patients receiving treatment within ”the golden hour” and leading to the improvement of functional outcomes at 90 days. Professor Fassbender's work has been instrumental in shaping contemporary approaches to diagnosing and managing stroke and neurodegenerative disease, making him a leading figure in modern neurology.

## Introduction and background

Today, neurology stands at the forefront of medical innovation, with ongoing research poised further to unravel the complexities of stroke and neurodegenerative disease, offering hope for more effective interventions and improved patient outcomes [1]. Professor Klaus Fassbender exemplifies these advances through his pioneering research regarding the molecular mechanisms of neurodegenerative diseases and stroke, leading to novel therapeutic strategies and improved clinical care [2-4]. His work bridges basic science and clinical application, directly contributing to the modern understanding and treatment of these complex neurological conditions. He pioneered the mobile stroke unit (MSU), which is a specialized ambulance equipped with a CT scanner, telemedicine capabilities, and clot-busting drugs, allowing for immediate diagnosis and treatment of stroke patients at the emergency site. Staffed by stroke-trained personnel, MSUs significantly reduce the time to treatment, improving patient outcomes by administering critical care directly in the field. This rapid response approach is crucial in minimizing brain damage during the "golden hour" following a stroke.

## Review

Early life and education

Klaus Fassbender was born in Germany, where he developed an early interest in medicine and neurology. He obtained his medical degree in 1989 from the University of Mainz. Professor Fassbender's extensive educational background includes studies in chemistry and medicine from 1980 to 1988, conducted at several distinguished institutions such as Wayne State University in Detroit, Università degli Studi di Padova in Italy, Johannes Gutenberg University in Mainz, and Rush Medical University in Chicago. He further specialized in rheumatology at the University Clinic Basel, Switzerland, from 1989 to December 1990, under the mentorship of Prof. Dr. W. Müller. From 1991 to 1992, he was a researcher at the Max Planck Institute for Psychiatry in Munich, guided by Prof. Dr. F. Holsboer. His specialist training culminated in the Department of Neurology Mannheim, Heidelberg University, from 1992 to 2002, where he worked under the guidance of Prof. Dr. M. Hennerici. From 2002 to 2004, he worked as a Professor and Vice Chairman in the Department of Neurology, University of Goettingen, under the direction of Professor M. Bähr.

Professor Fassbender completed his dissertation in 1990 on the topic "Otholith System in Movement" in the Department of Physiology, Johannes Gutenberg University in Mainz, Germany, under the supervision of Prof. Dr. R. v. Baumgarten. He later achieved his Habilitation in April 1997, focusing on "Pathomechanisms of Ischemic Neurodegeneration," conducted at the Department of Neurology Mannheim, Heidelberg University, under the guidance of Prof. Dr. M. Hennerici.

Career and contributions

Since December 2004, Professor Fassbender served as the Professor and Chairman of the Department of Neurology at Saarland University, Germany. Under his leadership, the department became a leading centre for neurological research and clinical practice in Germany. He was Vice President of the Saarland University from July 2007 to July 2009. In addition to his academic roles, he has held significant leadership positions, including heading the guideline commission for dementia of the German Neurological Society (DGN) from 2017 to 2019. Since 2006, he has been the President of the European Memory Clinic Association (EMCA). In 2008, he co-founded and has since co-directed the German Institute for Dementia Prevention. Furthermore, in 2017, he became the founding President of the Prehospital Stroke Treatment Organization (PRESTO).

Since 2000, Professor Fassbender has significantly contributed to neurological research, securing continuous grants from the German Research Foundation, BMBF, and other funding bodies. He served as a reviewer and as a member of the editorial boards of many major neurological and general medical journals, as well as an invited speaker at numerous international conferences. His achievements include the Research Award from the German Brain Association, the GenoPartal Health Care Award for dementia research, and the "Land der Ideen" Award in 2011 from the President of the Federal Republic of Germany. In addition, he coordinated the EU project MANAD (Microglial Activation in Alzheimer's Disease).

He published more than 370 peer-reviewed scientific articles. In addition to his research and clinical work, Professor Fassbender has been a dedicated educator and mentor to many aspiring neurologists. He supervised numerous doctoral theses and has been actively involved in teaching both at undergraduate and postgraduate levels. His commitment to education is reflected in his contributions to several textbooks and his role in organizing national and international conferences on neurology.

His translational research primarily focuses on stroke and neurodegenerative diseases of the nervous system.

Neuroinflammation and Dementia Research

Professor Fassbender's and his group's research significantly furthered our understanding of the disease pathogenesis using experimental autoimmune encephalomyelitis (EAE) models. He explored the inflammatory processes involved in multiple sclerosis, particularly the role of immune cells such as T-lymphocytes and their impact on neurodegeneration, thus contributing to a better understanding of how immune dysregulation leads to myelin damage and neuronal loss [5-11].

In Alzheimer's research, Professor Fassbender investigated the role of neuroinflammation and the impact of amyloid-beta and tau proteins in disease progression, contributing to a deeper understanding of the underlying mechanisms that drive cognitive decline [4,12-25]. For example, he elucidated the role of innate immune receptors on microglia, the mononuclear phagocyte of the brain, such as the LPS receptor and Toll-like receptors and their downstream intracellular signaling cascades, and stated the concept of structural mimicry between oligomeric amyloid peptide and complex components of microorganisms as a potential cause of chronic neuroinflammation and consecutive neurodegeneration in Alzheimer's disease [26,27].

Innovations in Stroke Treatment

**Figure 1:**
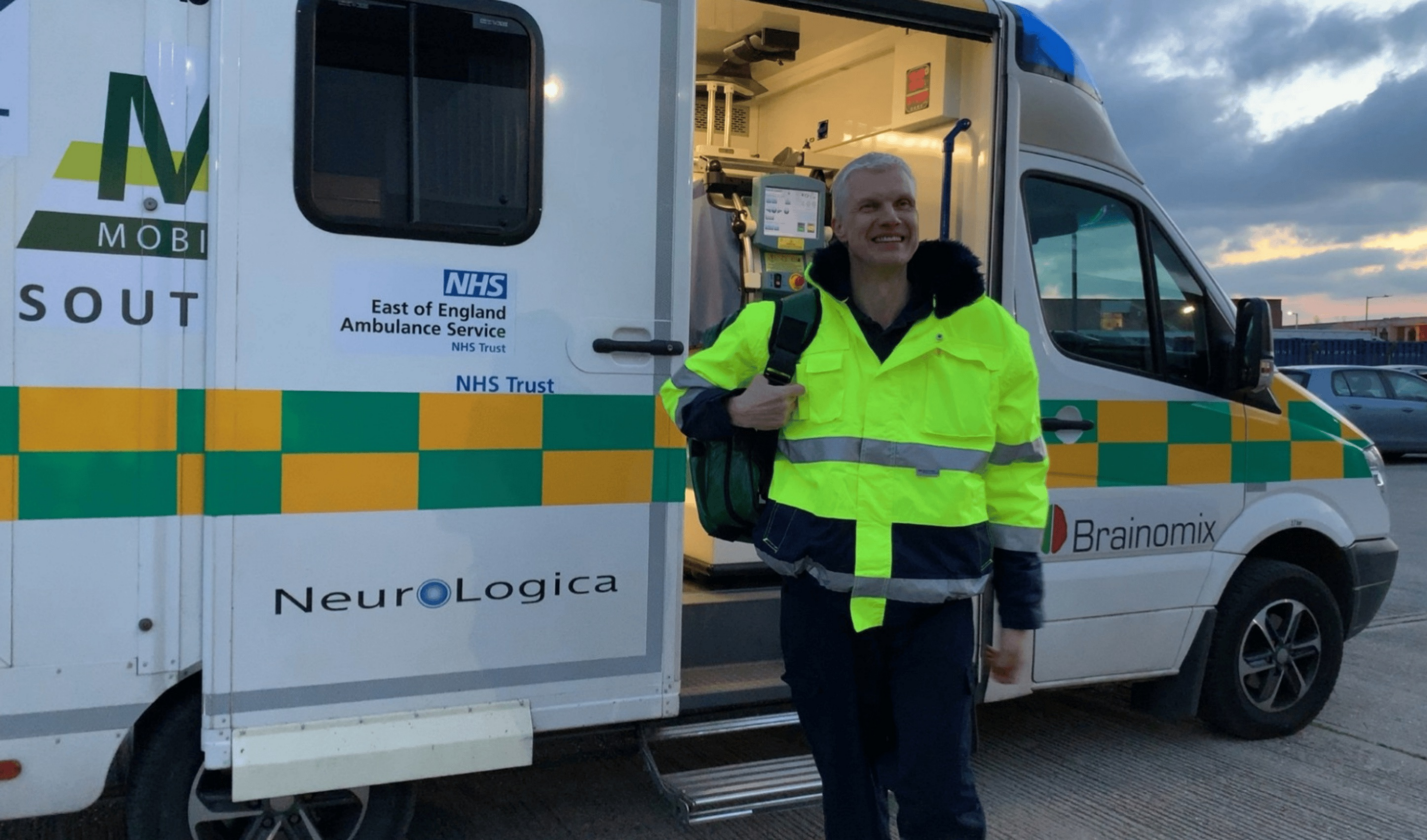
Professor Fassbender in front of a mobile stroke unit Southend-on-Sea, UK. December, 2018. Private image, written permission to publish obtained.

Professor Fassbender and his group pioneered the pre-hospital treatment of acute stroke. He first developed this approach in 2000 and, by 2008, had successfully integrated it into clinical care [28-33]. This innovation centers on the use of a specialized ambulance, the MSU, equipped with a multimodal CT scanner, a point-of-care laboratory, and essential medication. Through telemedicine, imaging results and video footage of the patient, and the examination are transmitted bidirectionally between the hospital and the ambulance [34-37], allowing for seamless integration with medical records and immediate specialist consultation [2,38-47]. This concept was further extended into acute pre-hospital care of urgent neurological conditions [41,48]. One of the obstacles encountered was cost-effectiveness in rural populations, where the incidence of stroke is lower. He came up with a novel solution and expanded the MSU to a hybrid MSU. This incorporates diagnostic devices such as X-ray, extended laboratory tests including drug screening, electroencephalogram (EEG), and ultrasound. This allows diagnosis and treatment of other acute emergencies such infections, sepsis, seizure, nonconvulsive status, and intoxication [49-51] (Fig. 1.)

In regard to in-hospital stroke management, the research of Professor Fassbender and his group has focused on process improvements within the hospital [52], including optimizing the "stroke room" [53,54] and integrating point-of-care laboratories [55], further reducing the time from hospital arrival to treatment initiation. His work on pre-hospital triage improved the identification and prioritization of stroke patients, ensuring that they receive appropriate care more swiftly. Professor Fassbender has also been instrumental in developing training guidelines for endovascular stroke therapy (EST) and interventional stroke treatments [56-62], contributing to the global standardization of these procedures [63].

Beyond acute care, Professor Fassbender's research has explored the impact of pre-existing conditions, such as age and pre-morbid factors [64-65] on stroke outcomes, and he has investigated the inflammatory response to ischemia [3,66-67], identifying potential biomarkers that could guide treatment decisions [68-74]. His work also addressed post-stroke complications [75], such as seizures [76-77], and the role of hemicraniectomy in managing severe cases [78]. These contributions have advanced the understanding of stroke pathophysiology and led to tangible improvements in patient care and outcomes, exemplifying the successful translation of laboratory research into clinical practice.

## Conclusions

Professor Fassbender has made transformative contributions to neurology. His groundbreaking work on the immune responses in Alzheimer's disease has paved the way for new treatments. Professor Fassbender development of the MSU represents a groundbreaking innovation in prehospital care, drastically reducing treatment times and improving outcomes for stroke patients. This achievement exemplifies Professor Fassbender's ability to translate complex molecular and cellular discoveries into practical, life-saving clinical applications. The MSU concept has now been adopted in numerous countries worldwide, notably in the US and Australia, and its use is implemented into the guidelines of the European Stroke Organisation. In addition, his leadership in neurological diseases along with his commitment to training and mentorship has shaped the modern neurological practice and influenced future generations of neurologists.
